# Impact of combination antiretroviral therapy initiation on adherence to antituberculosis treatment

**DOI:** 10.4102/sajhivmed.v16i1.346

**Published:** 2015-10-08

**Authors:** Marlene Knight, Robyn L. van Zyl, Ian Sanne, Jean Bassett, Annelies van Rie

**Affiliations:** 1 Clinical HIV Research Unit, University of the Witwatersrand, South Africa; 2 Department of Pharmacy and Pharmacology, University of the Witwatersrand, South Africa; 3 Right to Care, Johannesburg, South Africa; 4 Witkoppen Health and Welfare Centre, Johannesburg, South Africa; 5 Department of Epidemiology, University of North Carolina, United States

## Abstract

**Background:**

Healthcare workers are often reluctant to start combination antiretroviral therapy (ART) in patients receiving tuberculosis (TB) treatment because of the fear of high pill burden, immune reconstitution inflammatory syndrome, and side-effects.

**Object:**

To quantify changes in adherence to tuberculosis treatment following ART initiation.

**Design:**

A prospective observational cohort study of ART-naïve individuals with baseline CD4 count between 50 cells/mm^3^ and 350 cells/mm^3^ at start of TB treatment at a primary care clinic in Johannesburg, South Africa. Adherence to TB treatment was measured by pill count, self-report, and electronic Medication Event Monitoring System (eMEMS) before and after initiation of ART.

**Results:**

ART tended to negatively affect adherence to TB treatment, with an 8% – 10% decrease in the proportion of patients adherent according to pill count and an 18% – 22% decrease in the proportion of patients adherent according to eMEMS in the first month following ART initiation, independent of the cut-off used to define adherence (90%, 95% or 100%). Reasons for non-adherence were multifactorial, and employment was the only predictor for optimal adherence (adjusted odds ratio 4.11, 95% confidence interval 1.06–16.0).

**Conclusion:**

Adherence support in the period immediately following ART initiation could optimise treatment outcomes for people living with TB and HIV.

## Introduction

Adhering to a lengthy course of medication is difficult and poses a challenge to achieving health in people with chronic diseases. Poor adherence to treatment for infectious disease poses a risk to both the individual and community as it can lead to prolonged infectiousness, development of drug resistance, and poor treatment outcomes. Tuberculosis (TB) and the human immunodeficiency virus (HIV) present particular challenges as both are chronic diseases that mainly affect disadvantaged populations and involve complex treatment regimens with potentially severe side-effects.^1^ Treatment adherence for TB and HIV is also affected by beliefs about the origins and transmission of TB and HIV, which can result in stigmatisation of those affected.^2^

The 2012 World Health Organization (WHO) and 2015 South African antiretroviral therapy (ART) guidelines recommend initiating ART in people with TB as soon as possible, within the first 2 weeks of initiating TB treatment for those with profound immunosuppression (CD4 counts < 50 cells/mm^3^) and within the first 8 weeks of treatment in all TB patients.^3^ Whilst initiating ART greatly improves the survival and quality of life of TB patients living with HIV,^4^ it also poses challenges to patients and healthcare workers.^5^ Early initiation of ART can result in clinical deterioration related to immune reconstitution inflammatory syndrome (IRIS), toxic effects of drugs, or drug interactions, and increased pill burden. The high pill burden of four anti-TB drugs, antiretroviral drugs, and antimicrobial prophylaxis (cotrimoxazole and fluconazole) against opportunistic infections, as well as possible drug interactions and toxic effects, may jeopardise the patient's adherence to treatment.^6^ As a result, healthcare workers are often reluctant to start ART in patients receiving TB treatment. In 2012, only 57% of TB patients with HIV were started on ART,^7^ and in the latter group, initiation of ART was often delayed.^8^

In the present study, we aimed to quantify changes in adherence to TB treatment associated with initiation of ART by prospectively measuring adherence to TB drugs immediately before and after initiation of ART.

## Methods

### Study setting and population

The study took place at Witkoppen Health and Welfare Centre, a primary care clinic in Johannesburg, South Africa. Adults (> 18 years old) diagnosed with pulmonary TB who were ART-naïve at the time of initiation of TB treatment and had a CD4 count between 50 cells/mm^3 ^and 350 cells/mm^3^ were eligible for enrolment. Those with rifampicin-resistant TB (defined by Xpert MTB/RIF or culture-based drug susceptibility testing) were excluded as they are referred for care. Those with CD4 counts < 50 cells/mm^3^ were excluded because ART should be initiated as an emergency, limiting the possibility of reliably establishing the level of adherence to TB treatment before ART initiation. Those with CD4 counts > 350 cells/mm^3^ were excluded as these individuals were not eligible for ART, according to the 2010 South African Guidelines, which were current at the time of the study.

All care provision for TB and HIV, including the decision on timing of ART initiation, was performed by the routine clinic staff, without any input from study staff.

### Study procedures

Eligible patients who signed informed consent completed a questionnaire collecting information on socio-demographic information, occurrence of side-effects, and adherence support. Medical files were reviewed to collect details of weight, height, results of TB diagnostics (Xpert MTB/RIF, smear microscopy and culture), TB treatment outcome, ART regimen and start date, and CD4 count and viral load (VL) at baseline and during the first 6–12 months of ART.

Ethical approval was obtained from the Institutional Review Board of the University of North Carolina (10–2317) and the University of the Witwatersrand's Human Research Ethics Committee (M10925).

### Adherence measures

Participants were prospectively monitored for adherence, using pill count (primary measure of adherence), self-report, and an electronic Medication Event Monitoring System (eMEMS). Participants received their TB medication in a Securitainer fitted with an eMEMS lid (eMuM, GeoMed, Stellenbosch, South Africa). Participants were seen by study staff at each clinic visit; the number of visits varied and was determined by the routine clinic care provider. At each visit, the date of visit, number of pills distributed for TB treatment, and prescribed dosage were recorded. At each return visit, the number of pills remaining in the container was recorded, the eMEMS lid was connected to a computer to download data pertaining to when the Securitainer was opened, and the patient was asked, ‘In the last week, have you missed any of your doses?’ Participants with suboptimal adherence (according to self-report, pill count or eMEMS) were asked the reasons for non-adherence.

To reduce the effect of factors other than ART initiation on adherence to TB treatment, the primary outcome measure was adherence during the 28 days before and 28 days after ART initiation. The number of days included varied by participant because ART could be initiated sooner than 28 days after starting TB treatment, and the number of days between visits was not always exactly 28 days.

Pill count was used to calculate several adherence measures: percentage of the prescribed doses taken (100 × [number of pills dispensed minus the number of pills returned]/[number of days between clinic visits] × [daily dose]) and three binary indicators: whether adherence equalled 100%, ≥ 95% and ≥ 90%. eMEMS data were used to calculate additional measures of adherence: percentage of the prescribed doses taken (100 × number of days with bottle openings/number of days between clinic visits) and three binary indicators: whether adherence was 100%, ≥ 95% and ≥ 90%. The number of daily bottle openings were truncated to one to avoid overestimating adherence. Data from follow-up visit questionnaires were used to calculate the self-reported adherence (100 × [1 minus the number of missed doses]/number of days between clinic visits) and three binary indicators: whether adherence was 100%, ≥ 95% and ≥ 90%.

### Statistical analysis

Participant characteristics are presented as absolute and relative frequencies for categorical variables and as medians and interquartile ranges (IQR) for continuous variables.

The effect of ART initiation on adherence to TB treatment was evaluated in two different ways. Firstly, the median change in percentage of prescribed doses of TB drugs taken before and after ART initiation was compared for each of the three continuous adherence measures (pill count, eMEMS and self-report) using the Wilcoxon matched-pair signed ranks test. Secondly, using the exact McNemar test for paired samples, the proportion of participants adherent to TB treatment before and after ART initiation was compared for all three binary adherence measures for each of the three methods (pill count, eMEMS and self-report).

To determine factors predictive of optimal adherence (100% adherence) in the first month after ART initiation, we first performed bivariate analysis to estimate crude odds ratios (OR) and 95% confidence intervals (CI). We subsequently ran a saturated logistic model containing all selected covariates to estimate adjusted ORs (aOR), and used stepwise backwards elimination to generate a final (reduced) predictive model. We conducted a sensitivity analysis to explore the effect of broadening the definition of optimal adherence to ≥ 95% and ≥ 90% adherence post ART. Crude ORs and aORs are presented with standard Wald 95% CI. All analyses were conducted using STATA 12.1 (Texas, USA).

## Results

### Study cohort characteristics

**FIGURE 1:**
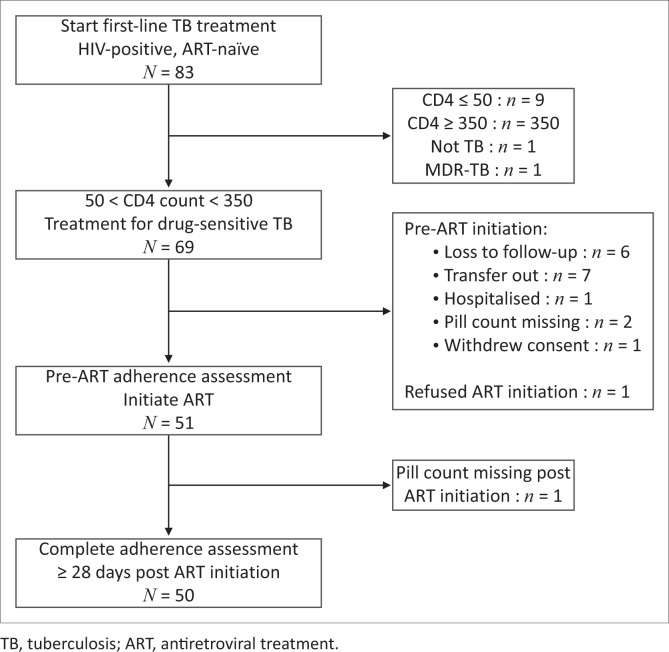
Study flow chart.

Between September 2011 and October 2012, 83 ART-naïve individuals initiating first-line drugs for pulmonary TB gave informed consent for study participation (Figure 1). Of these, 14 were excluded from the analysis for not meeting all inclusion criteria: CD4 count < 50 cells/mm^3^ (*n* = 9), CD4 count > 350 cells/mm^3^ (*n* = 3), erroneous TB diagnosis (*n* = 1), or MDR-TB diagnosis (*n* = 1). Prior to ART initiation, another 18 were excluded owing to loss to follow-up (*n* = 6), transfer to another facility (*n* = 7), hospitalisation (*n* = 1), refusal to start ART (*n* = 1), missing pill count (*n* = 2), and withdrawal of consent (*n* = 1). One additional participant was excluded because of missing pill count post ART initiation.

Among the 50 patients included in the analysis, median age was 32.5 years (IQR 30–38), 56% were female, 58% were unemployed, and almost half (48%) were not of South African nationality. The diagnosis of TB was bacteriologically confirmed in 80%. Median CD4 count was 124 (IQR 94–193). The median time to ART initiation was 27 days (IQR 13–48), and almost all (92%) participants initiated an ART regimen containing efavirenz, lamivudine and tenofovir. TB treatment outcome was successful in 82%. Only 56% of participants achieved viral load suppression, defined as a viral load < 400 copies/mL in the 6–12 months of ART (Table 1).

**TABLE 1:** Characteristics of 50 individuals included in the analysis of the impact of initiation of antiretroviral treatment on adherence to tuberculosis treatment.

Continuous variables	Group	Median *n*	IQR %
Gender	Male	22	44.0
	Female	28	56.0
Employed	Yes	21	42.0
	No	29	58.0
Nationality	South Africa	26	52.0
	Zimbabwe	21	42.0
	Mozambique	3	6.0
Education†	None	2	4.1
	Primary school	4	8.2
	Some secondary school	28	57.1
	Secondary school completed	15	30.6
TB diagnosis	Confirmed	40	80.0
	Clinical	10	20.0
ART regimen	EFV, 3TC, TDF	46	92.0
	EFV, 3TC, D4T	3	6.0
	EFV, 3TC, AZT	1	2.0
TB treatment outcome	Cure	9	18.0
	Treatment completed	32	64.0
	Treatment failed	0	0.0
	Died	1	2.0
	Lost to follow-up	5	10.0
	Not evaluated (transfer out)	3	6.0
ART outcome	Suppressed‡	28	56.0
	Failure to suppress‡	10	20.0
	Died	1	2.0
	Lost to follow-up	8	16.0
	Not evaluated (transfer out)	3	6.0
**Age (years)**		**32.5**	**30–38**
**BMI at enrollment**		**21.5**	**19–23**
**CD4 count at enrollment (cells/mm**^3^**)**		**124**	**94–193**
**Time from TB treatment to ART initiation (days)**		**27**	**13–48**

BMI, body mass index; TB, tuberculosis; ART, antiretroviral treatment; IQR, interquartile ranges.

†, Education level missing for 1 participant; ‡, suppression is defined as viral load < 400 copies/mL within the first year of ART.

### Adherence to TB treatment before and after antiretroviral therapy initiation

When measured by pill count or self-report, the median percentage of prescribed TB drug doses taken was 100% before and after ART initiation (Table 2). When measured by eMEMS, median percentage of prescribed TB drug doses taken was 93% before, and 82.5% (IQR 55–96) after, ART initiation. There was no change in the percentage of TB medication taken before and after ART initiation, with a median percentage change of 0 (IQR −4, +1) for pill count, 0 (IQR 0–0) for self-report, and minus 1.5 (IQR −25, +3) for eMEMS (all *p* values > 0.30).

**TABLE 2a:** Adherence to tuberculosis treatment before and after initiation of antiretroviral treatment.

Adherence measure	% TB drugs prescribed taken before ART initiation†			% TB drugs prescribed taken after ART initiation‡			% change in TB drugs taken before and after ART initiation			
		*N*	Median	IQR	*N*	Median	IQR	*N*	Median	IQR	*p* value§
Pill count	50	100	96, 100	50	100	91, 100	50	0	−4, +1	0.55
Self-report	50	100	100, 100	50	100	100, 100	50	0	0, 0	0.64
Electronic MEMS	21	93	75, 100	20	82.5	55, 96	14	−1.5	−25, +3	0.31

MEMS, Medication Event Monitoring System; TB, tuberculosis; ART, antiretroviral treatment; IQR, interquartile ranges.

†, Period assessed is the period up to 28 days pre ART initiation; ‡, Period assessed is the 28-day period following ART initiation. If the first TB clinic visit occurred > 28 days following ART initiation, the entire time period between ART initiation and subsequent TB clinic visit was included; §, Comparison only possible between 14 patients who had MEMS data available both before and after ART initiation.

**TABLE 2b:** Hundred percent adherence to tuberculosis treatment before and after initiation of antiretroviral treatment.

Adherence measure	Proportion of patients with 100% adherence pre ART			Proportion of patients with 100% adherence post ART			Difference in proportion adherent post v. pre cART (%)	*p* value‡
		*N*	%	95% CI	*N*	%			95% CI		
Pill count	50	64.0	50.2–77.8	0.50	56.0	41.7–70.3	−8.0	0.50
Self-report	50	96.0	90.4–100	1.00	94.0	87.2–100	−2.0	1.00
Electronic MEMS	21	38.1	15.4–60.7	0.5†	10.0	0.4–24.4	−18.1	0.5†

MEMS, Medication Event Monitoring System; ART, antiretroviral treatment; CI, confidence intervals.

†, Wilcoxon matched-pair signed ranks test; ‡, Exact McNemar test for paired samples.

**TABLE 2c:** Ninety-five percent adherence to tuberculosis treatment before and after initiation of antiretroviral treatment.

Adherence measure	Proportion of patients with ≥ 95% adherence pre ART			Proportion of patients with ≥ 95% adherence post ART			Difference in proportion adherent post v. pre cART (%)	*p* value‡
		*N*	%	95% CI	*N*	%			95% CI		
Pill count	50	78.0	66.1–89.9	0.36	68.0	54.6–81.4	−10.0	0.36
Self-report	50	100	100–100	1.00	98	94.0–100	−2.0	1.00
Electronic MEMS	21	47.6	24.3–70.9	1.00†	30.0	8.0–52.0	−17.6	1.00†

MEMS, Medication Event Monitoring System; ART, antiretroviral treatment; CI, confidence intervals.

†, Wilcoxon matched-pair signed ranks test; ‡, Exact McNemar test for paired samples.

**TABLE 2d:** Ninety percent adherence to tuberculosis treatment before and after initiation of antiretroviral treatment.

Adherence measure	Proportion of patients with ≥ 90% adherence pre ART			Proportion of patients with ≥ 90% adherence post ART			Difference in proportion adherent post v. pre cART (%)	*p* value‡
		*N*	%	95% CI	*N*	%			95% CI		
Pill count	50	88.0	78.7–97.3	0.18	78.0	66.1–89.9	−10.0	0.18
Self-report	50	100	100–100	1.0	100	92.9–100	0	1.0
Electronic MEMS	21	61.9	39.3–84.6	0.63†	40.0	16.5–63.5	−21.9	0.63†

MEMS, Medication Event Monitoring System; ART, antiretroviral treatment; CI, confidence intervals.

†, Wilcoxon matched-pair signed ranks test; ‡, Exact McNemar test for paired samples.

When measured by pill count, the proportion of participants who were 100% adherent to TB treatment before and after ART initiation was 64% (41.7–70.3) versus 56.0% (50.2–77.8); increased to 68.0 (54.6–81.4) versus 78% (66.1–89.9) when adherence was defined as 95% of prescribed doses taken, and 78.0 (66.1–89.9) versus 88.0% (78.7–97.3) when adherence was defined as 90% of doses taken.

Self-reported adherence was high, with 100% of participants being adherent before and after ART initiation when adherence was defined as taking ≥ 90 or ≥ 95% of prescribed doses, and 96.0% (90.4–100) and 94.0% (87.2–100) being adherent before and after ART initiation, respectively, when adherence was defined as 100% of prescribes doses.

Owing to technical errors, power failures, equipment failures, and misunderstandings by pharmacists and patients, adherence data by eMEMS were only available for 21 participants pre ART, 20 participants post ART, and 14 both before and after ART initiation. The proportion of participants adherent to TB treatment after ART initiation was consistently lower than adherence before ART initiation: 10.0% (0.4–24.4) versus 38.1 (15.4–60.7); 30.0 (8.0–52.0) versus 47.6 (24.3–70.9) and 40.0 (16.5–63.5) versus 61.9 (39.3–84.6) when adherence was defined as 100%, 95% or 90% of prescribed doses taken, respectively.

### Reasons for suboptimal adherence

Leaving house without tablets (*n* = 8) and running out of tablets between visits (*n* = 8) were the most frequently stated reasons for missing doses (*n* = 8), followed by forgetfulness (*n* = 3), dosing errors (*n* = 3), taking medication as prescribed whilst not using the eMEMS lid (*n* = 3), distractions (*n* = 2), medication side-effects (*n* = 2) and lack of transport money (*n* = 1).

### Factors associated with optimal adherence to TB treatment after antiretroviral therapy initiation

Employment status was the only factor associated with optimal (100%) adherence following ART initiation. Compared with those not employed, participants who were employed had four times greater odds (aOR 4.11, 95% CI 1.06–16.0) of being fully adherent to TB treatment. Age and gender tended to be associated with optimal adherence, with a 13% (95% CI −1, +29%) increased odds for every year increase in age and men being less likely to optimally adhere to TB treatment (aOR 0.32, 95% CI 0.08–1.29), but these associations did not reach statistical significance. In sensitivity analyses, age and employment status were factors associated with 95% and 90% adherence, respectively (Table 3).

**TABLE 3:** Factors associated with optimal adherence (100% of prescribed doses) following initiation of antiretroviral treatment in patients receiving treatment for active tuberculosis (pe 0.05; pr 0.15).

Factor	Characteristic	Crude OR (95% CI)	Adjusted OR full model (95% CI)	Adjusted OR final model (95% CI)
Age	Per year increase	1.12 (1.00–1.25)	1.11 (0.97–1.28)	1.13 (0.99–1.29)
Gender	Female	Referent	Referent	Referent
	Male	0.80 (0.26–2.45)	0.34 (0.60–1.95)	0.32 (0.08–1.29)
Nationality	South African	Referent	Referent	-
	Not South African	0.83 (0.27–2.55)	1.48 (0.30–7.14)	-
Education	Secondary school not complete	Referent	Referent	-
	Secondary school completed	0.36 (0.10–1.27)	0.37 (0.07–1.91)	-
Employment	Unemployed	Referent	Referent	Referent
	Employed	4.53 (1.30–15.77)	3.87 (0.80–18.8)	4.11 (1.06–16.0)
CD4 count	(per 100 cells/mm^3^ decrease)	1.37 (0.61–3.00)	1.19 (0.43–3.30)	-
BMI	Per unit increase	0.98 (0.85–1.14)	1.01 (0.92–1.11)	-
Time between TB treatment and ART initiation	Per day increase	1.01 (0.98–1.02)	1.01 (0.98–1.04)	-
Adherence to TB treatment before ART initiation	< 100%	Referent	Referent	-
	100%	2.08 (0.64–6.73)	1.40 (0.28–6.87)	-
Family DOT post ART	No	Referent	Referent	-
	Yes	0.40 (0.12–1.25)	0.81 (0.16–4 .06)	-

BMI, body mass index; TB, tuberculosis; ART, antiretroviral treatment; DOT, directly observed treatment; OR, odds ratios; CI, confidence intervals.

## Discussion

In the present study of ART-naïve HIV-infected individuals receiving treatment for active TB, we observed a trend of decrease in adherence to TB treatment in the first month following ART initiation, with an 8% – 10% decrease in the proportion of patients adherent according to pill count and an 18% – 22% decrease in the proportion of patients adherent according to eMEMS, independent of the cut-off used to define adherence (90%, 95% or 100% of prescribed doses taken). The finding of reduced adherence soon after the introduction of ART is clinically relevant, as suboptimal adherence has been associated with poor treatment outcomes and development of resistance, especially in the early phases of treatment when the bacillary load is highest. Whilst many have speculated that the increased pill count in patients with TB initiating ART could reduce adherence,^5,6,9^ we could not compare our findings with others as we could not find published reports assessing this association.

Similar to findings of other studies,^2^ reasons for non-adherence reported by patients were multifactorial and few independent predictors for optimal adherence could be identified. Except for employment, with those being employed having four times higher odds of remaining fully adherent when initiating ART, we could not identify patient factors associated with adherence.

In the present study, we used three different methods to measure adherence: self-report of missed doses, pill count, and eMEMS. We observed that adherence by self-report was always highest, pill count gave intermediate estimates, and eMEMS consistently resulted in the lowest estimates. Poor correlations between different adherence measures have been reported. For example, in a study of adherence to ART, Holzemer found that there was minimal correlation amongst adherence as measured by pharmacy refill, self-report, MEMS and pill count.^10^ Overestimation of adherence by self-report is a consistent finding, probably related to social desirability or recall error.^11^ eMEMS, on the other hand, can underestimate adherence when several doses of medications are removed from bottles at a single time, as was observed in the present and other studies.^12,13^ Similar to what was observed in our cohort, self-reported rates of adherence are higher than the rates derived from electronic monitoring; however, the 40% – 50% difference between the two measures is greater than the 10% – 30% reported in other settings.^14,15^ This result may be owing to limited validity of the eMEMS data, given the numerous challenges when implementing eMEMS into routine care in a resource-limited setting, including batteries of eMEMS caps running flat, power cuts during the transfer of eMEMS data to computer, errors made by pharmacists when filling the containers, and breakage of the container lids. In addition, some patients forgot or lost their Securitainer.

The present study has several limitations. Firstly, the small sample resulted in imprecise estimates and a lack of power to detect statistically significant differences, even when the differences observed were probably of clinical relevance. Secondly, the time between starting TB treatment and ART initiation in the study participants was short, (median of 27 days), and did not vary greatly between participants (IQR 13–48). As such, we could not assess the impact of timing of ART initiation on adherence. It is possible that delay of ART initiation until the end of the intensive phase, when patients feel better and side-effects of TB treatment have subsided, could lower the negative impact of ART initiation on adherence to TB treatment. This possibility would need to be weighed against the risk of poor treatment outcomes owing to the delay of ART initiation, and may therefore only be possible for patients with high CD4 counts at the time of TB diagnosis. Thirdly, we limited our assessment to the 28 days immediately before and after ART initiation to assess the impact of ART initiation on adherence to TB drugs. We could therefore not assess whether the observed changes in adherence were temporary or persisted throughout the TB treatment period. Finally, the clinic did not perform clinic-based directly observed treatment (DOT). Findings of the present study may therefore not be generalisable to settings where DOT is systematically implemented for all patients receiving TB treatment.

## Conclusion

In the present small prospective cohort study, we observed a trend to decreased adherence to TB treatment following the initiation of ART. Our findings suggest that adherence interventions in the period following ART initiation may be needed to optimise treatment outcomes for people living with TB and HIV.
